# Sequence-dependent DNA helical rise and nucleosome stability

**DOI:** 10.1186/1471-2199-10-105

**Published:** 2009-11-27

**Authors:** Francesco Pedone, Daniele Santoni

**Affiliations:** 1 Dept. of Genetics and Molecular Biology, 'Sapienza' University, P.le A. Moro 3, 00161 Rome, Italy

## Abstract

**Background:**

Nucleosomes are the basic structural units of eukaryotic chromatin and play a key role in regulation of gene expression. After resolution of the nucleosome structure, the bipartite nature of this particle has revealed itself and has disclosed the presence, on the histone surface, of a symmetric distribution of positive charges, able to interact with their negative DNA phosphate counterpart.

**Results:**

We analyzed helical steps in known nucleosomal DNA sequences, observing a significant relationship between their symmetric distribution and nucleosome stability. Synthetic DNA sequences able to form stable nucleosomes were used to compare distances on the left and on the right side of the nucleosomal dyad axis, where DNA phosphates and charged residues of the (H3H4)_2_-tetramer interact. We observed a linear relationship between coincidence of distances and nucleosome stability, i. e., the more symmetric these distances the more stable the nucleosome.

**Conclusion:**

Curves related to this symmetric distribution along the DNA sequence identify preferential sites for positioning of the dyad axis, which we termed *palinstases*. The comparison of our data with known nucleosome positions in archaeal and eukaryotic sequences shows many coincidences of location. Sequences that impair nucleosome formation and DNase I hypersensitive sites yield curves with a lower degree of symmetry. Analysis performed on DNA tracts of promoters close to the transcription start and termination sites identified peculiar patterns: in particular low affinity for nucleosome binding at the transcription start site and a high affinity exactly at the transcription termination site, suggesting a major role of nucleosomes in the termination of transcription.

## Background

The role played by the DNA sequence in determining preferred positions of individual nucleosomes has been studied using both experimental and theoretical approaches. Several global assessments of nucleosome positioning have been described in yeast [1-4], in *Caenorhabditis elegans *[5,6], in *Drosophila *[7] and in humans [8-13]. Experimental mapping of nucleosomes has been performed mainly by micrococcal nuclease digestion followed either by ligation-mediated PCR analysis or by DNA microarray-based methods. Theoretical models used for nucleosome-positioning prediction include probabilistic models [1], the comparative genomics approach [14], the support vector machine classifier [3], energy landscapes [15] and DNA physical properties [16]. During nucleosome formation, 60 bp in the central region of nucleosomal DNA become primarily associated with (H3H4)_2_-tetramer [17]. The histone particle presents, on its surface, a distribution of positive charges able to interact with their negative DNA phosphate counterpart. These charges are symmetrically distributed with respect to the pseudo-dyad axis of the nucleosome and constitute a 'mask' of distances that remained constant during evolution [18]. It is usually assumed that DNA length is the same for any DNA sequence of the same size and that the helical rise of any dinucleotide step does not shift to a large extent from the mean value of about 3.4 Å. More recent results, obtained by X-ray analysis of DNA crystals, suggest helical rise values around 2.83 ± 0.36 Å for A-DNA and 3.29 ± 0.21 Å for B-DNA [19]. We observed that DNA oligomers having the same number of base pairs, as reported in X-ray and NMR databases, show different lengths, i.e., the length of dodecamers varies from 32 up to 37Å.

We guessed that nucleosome positioning must be related to a symmetric distribution of distances along the DNA sequence upstream and downstream of the presumed dyad-axis location. In order to measure the length of DNA sequences, as the sum of helical steps, we have collected from literature available helical rise values of the 136 possible tetranucleotide steps of DNA.

## Results and Discussion

### Helical rise values of tetranucleotides

A tetranucleotide code for the 136 possible tetrads was obtained collecting data from available databases of resolved DNA structures (see Methods, table 1). We referred each value to the central dinucleotide helical step, taking into account the first two flanking bases. For instance, we assigned a value of 3.40 Å to ACTG tetrad, meaning that the central dinucleotide CT has such a value if it occurs with an A and a G as flanking bases.

**Table 1:** Helical rise values of the 136 possible tetranucleotides.

N°	Tetranuc	h r (Å)	rmsd (Å)	Origin of data	N°	Tetranuc	h r (Å)	rmsd (Å)	O**rigin of data**
1	AAAA\TTTT	3.21	0.19	1fzx.1nev	69	TCCC\GGGA	4.20	----	1kbd
2	AAAG\CTTT	3.17	0.09	1cs2,1kbd,3kbd	70	CCCC\GGGG	4.37	----	1kbd
3	AAAC\GTTT	3.05	0.22	1fzx,1g14,1nev	71	GCCC\GGGC	2.81	0.51	*ad0002, ad0003, ad0004, adh008*
4	AAAT\ATTT	3.38	----	*bdl038*	72	ACCC\GGGT	3.32	----	mean CC/GG
5	TTTC\GAAA	3.07	0.01	1g14,1kbd,3kbd	73	CGGA\TCCG	3.15	0.20	1agh,1la1
6	CTTC\GAAG	3.04	0.05	1agh,1axp	74	CCCG\CGGG	3.03	0.26	*adh008, adh0102, adh0103, adh0105*
7	GAAC\GTTC	3.12	----	mean AA/TT	75	CGGC\GCCG	2.91	----	1uqf
8	ATTC\GAAT	3.36	0.12	1bn9,1bwt,1gip,2dau	76	ACCG\CGGT	3.23	0.04	1 d20,1la1
9	CAAA\TTTG	2.89	0.01	1fzx,1nev	77	TCCA\TGGA	3.33	0.28	1kbd,3kbd
10	CAAG\CTTG	2.98	0.12	140d,141d,142d,1agh,1g14	78	CCCA\TGGG	3.48	----	1kbd
11	CAAC\GTTG	3.03	----	1bdz,	79	TGGC\GCCA	2.94	----	1but
12	ATTG\CAAT	3.27	----	1buf,	80	ACCA\TGGT	3.37	----	1afz
13	TAAA\TTTA	3.08	----	1cs2	81	ACAA\TTGT	2.89	0.58	1agh,1bdz
14	CTTA\TAAG	3.12	----	mean AA/TT	82	ACAG\CTGT	2.99	----	mean CA/TG
15	TAAC\GTTA	3.27	----	1cqo,	83	ACAC\GTGT	3.19	----	1la1
16	ATTA\TAAT	2.88	----	1 d70	84	ACAT\ATGT	2.61	0.39	1bn9,1saa,
17	AAGA\TCTT	3.17	0.11	1agh,1axp,1g14	85	TTGC\GCAA	2.74	0.09	140d,141d,142d,1fzx,1g14,1nev
18	AAGG\CCTT	2.97	0.03	140d,141d,142d	86	CTGC\GCAG	3.73	0.58	1afz,1bdz,1cs2,1opq,3kbd
19	AAGC\GCTT	3.20	0.07	140d,141d,142d,1axp,1cs2	87	GCAC\GTGC	2.86	0.14	*adh047, adj075*
20	AAGT\ACTT	3.77	0.41	1kbd,3kbd	88	ATGC\GCAT	3.10	0.33	1 d18,1skp
21	TCTC\GAGA	3.19	0.19	1axp,1opq	89	CCAA\TTGG	3.78	0.05	**1g5d.1g5e,1giz,1gj0**
22	CCTC\GAGG	3.24	----	mean CT/AG	90	CCAG\CTGG	3.19	0.32	1kbd,3kbd
23	GAGC\GCTC	2.80	0.44	1k9h,1k9l,3kbd	91	CCAC\GTGG	2.86	1.00	1afz,1kkv
24	ACTC\GAGT	2.95	----	1dk9	92	ATGG\CCAT	2.85	----	1but
25	CAGA\TCTG	3.92	----	1opq	93	TCAA\TTGA	2.66	0.12	140d,141d,142d
26	CAGG\CCTG	3.09	0.13	1afz,1kbd,3kbd	94	CTGA\TCAG	3.06	----	1bn9
27	CAGC\GCTG	3.39	----	1uqb	95	TCAC\GTGA	3.01	0.09	1 d20,1k9h,1saa,3kbd
28	ACTG\CAGT	3.40	0.17	1bdz,1cs2	96	ATGA\TCAT	2.36	0.26	1dk9,1k9l,1skp
29	TAGA\TCTA	3.21	0.33	1bn9,1 d20	97	AATA\TATT	3.23	----	mean AT/AT
30	CCTA\TAGG	3.26	----	1bn9	98	AATG\CATT	3.34	0.01	1bn9,1d70
31	TAGC\GCTA	2.96	----	1g7z	99	AATC\GATT	3.23	----	mean AT/AT
32	ACTA\TAGT	3.29	----	*bdj061*	100	AATT	3.43	0.14	1buf,1bwt,1gip,2dau
33	AACA\TGTT	3.41	----	mean AC/GT	101	TATC\GATA	3.16	0.19	1 d20,1skp
34	AACG\CGTT	3.42	0.31	1cqo,1fzx,1g14,1nev	102	CATC\GATG	3.23	----	mean AT/AT
35	AACC\GGTT	3.41	----	mean AC/GT	103	GATC	3.22	0.16	1opq,1uqd
36	AACT\AGTT	3.56	----	1bdz	104	CATA\TATG	3.31	0.24	1k9l,1skp,1uqa
37	TGTC\GACA	3.22	0.06	1agh,1la1	105	CATG	3.20	0.23	1but,1d18,1dk9,1saa
38	CGTC\GACG	3.90	----	1saa	106	TATA	2.94	0.12	1d42,1d70
39	GACC\GGTC	3.57	----	1bn9	107	AGCA\TGCT	3.53	0.05	1cs2,3kbd
40	AGTC\GACT	3.66	----	1kbd	108	AGCG\CGCT	3.42	0.09	1q7z,1k9h,1k9l
41	CACA\TGTG	3.30	0.17	1k9h,1saa	109	AGCC\GGCT	3.44	----	**214d**
42	CACG\CGTG	3.46	0.10	1kkv,1uqc	110	AGCT	3.29	0.07	140d,141d,142d,1uqb
43	CACC\GGTG	3.00	0.24	1afz,1 d20,1la1	111	TGCC\GGCA	3.08	0.20	140d,141d,142d,1afz,1fzx,1g14,1nev
44	AGTG\CACT	3.58	----	3kbd	112	CGCC\GGCG	3.16	0.18	**1a9g,1a9h**
45	TACA\TGTA	3.14	----	1bn9	113	GGCC	3.40	0.25	1but,1uqf
46	CGTA\TACG	3.25	0.19	1 d19,1 d68,1g80.1uqe	114	CGCA\TGCG	4.46	----	1opq
47	TACC\GGTA	3.26	0.21	**1axu,1bw7**	115	CGCG	3.35	0.36	1gip,1kkv,1la1,1uqq,1dau
48	AGTA\TACT	3.42	0.05	1cs2,1dk9	116	TGCA	3.63	0.20	1bdz,1d18
49	AGAA\TTCT	3.15	0.12	xp,1bn9,1g14	117	ACGA\TCGT	3.12	0.07	**1onm,1sot**
50	AGAG\CTCT	3.01	0.25	1axp1agh,1a	118	ACGG\CCGT	2.81	0.49	1fzx,1g14,1nev
51	AGAC\GTCT	3.08	----	*adh041*	119	ACGC\GCGT	2.48	0.36	1cqo,1d68,1q80,1kkv
52	ATCT\AGAT	3.02	----	1opq	120	ACGT	4.02	1.35	1d19,1saa,1uqc
53.	TTCC\GGAA	3.38	0.19	1kbd,3kbd	121	TCGC\GCGA	3.36	0.16	1bwt,1gip,1opq,2dau
54	CTCC\GGAG	3.24	----	mean AG/TC	122	CCGC\GCGG	2.83	----	1la1
55	GTCC\GGAC	3.46	0.04	1agh,1kbd,1la1	123	GCGC	2.61	0.22	1k9h,1k9l,1uqq
56	ATCC\GGAT	3.24	----	mean GA/TC	124	CCGA\TCGG	3.33	----	*bd0001*
57	CGAA\TTCG	3.15	0.31	1bwt,1gip,2dau	125	CCGG	2.85	0.33	*adh008, adj022*
58	CTCG\CGAG	3.98	----	1opq	126	TCGA	3.44	0.19	*bdj025, bdj060*
59	CGAC\GTCG	3.34	0.16	**1onm,1rn9,1s74,1sot**	127	ATAA\TTAT	2.69	----	1 d70
60	ATCG\CGAT	3.29	----	1uqd,	128	ATAG/CTAT	2.63	0.65	1 d20,1skp
61	TTCA\TGAA	3.24	0.11	**1hm1,1onm**	129	ATAC\GTAT	2.72	0.19	1d42,1d68,1d70
62	CTCA\TGAG	3.03	0.17	140d,141d,142d,1dk9,1k9h,1k9l,3kbd	130	ATAT	2.47	0.80	1d42,1skp,1uqa
63	TGAC\GTCA	3.10	0.18	1bn9,1saa	131	TTAC\GTAA	2.62	0.35	**1bid,1bw7**
64	ATCA\TGAT	3.18	0.08	1 d20,1skp,	132	CTAC\GTAG	2.86	0.09	1bn9,1cs2,
65	AGGA\TCCT	3.32	----	mean CC/GG	133	GTAC	2.90	0.27	1d19,1dk9,1g80,1uqe
66	AGGG\CCCT	2.96	0.12	*ad0003, ad0004, adh078*	134	CTAA\TTAG	2.60	----	1cs2
67	AGGC\GCCT	2.92	0.07	140d,141d,142d	135	CTAG	2.94	----	1g7z
68	ACCT\AGGT	3.82	0.30	1afz,1bn9	136	TTAA	3.21	----	1cqo

Columns report from the left to the right: sample number, sequence/reverse complement of the tetranucleotide, helical rise in angstrom units, root mean square deviation of the helical rise, name of samples used to retrieve data from Nucleic Acid Database at http://ndbserver.rutgers.edu/atlas. Names in normal characters are from NMR database of DNA duplexes, names in boldface are from NMR database for DNA duplexes accommodating a modified base and names in italics are from X-ray database.

Data reported in table 1 show a distribution of helical rise values with a mean of 3.2 Å and a maximal and a minimal value of 4.46 Å (step 114 CGCA/TGCG) and 2.36 Å (step 96 ATGA/TCAT), respectively, with a remarkable difference of 2.1 Å between these two values. Thirteen of the values reported in the table were calculated by averaging values for tetranucleotides containing the same central dinucleotide step. For these tetranucleotides and 39 additional ones, whose helical rise values were derived by a single DNA oligomer, rmsd values are absent. Therefore, a refinement of the table is needed using new available resolvedstructures.

It is remarkable that tetranucleotides whose rmsd values are higher than 0.3 Å have central dinucleotides that can be stacked, in the DNA helix, into two different conformations; that's why they are termed 'bistable'. Hunter [20] reports evidence of bistability in DNA bp, mainly in the pyrimidine-purine CG and TA steps, but also in CC/GG and AG/CT. A re-classification of bistability was performed by Gardiner et al. [21] in a study on structural parameters of DNA oligomers, and, in tetranucleotides, the bistability turned out to be dependent of the central step according to GG, CG, CA > GC > TA > AG, GA, AC, AT, AA order. Therefore, we conclude that high variability of helical rise values for some of the tetranucleotides in table 1 is due to the presence of a central bistable dinucleotide step, which exhibits a high sensitivity to neighboring base pairs.

Lu and Olson [19] have shown that the variation of helical rise values in dinucleotide steps is related to coupling of roll and slide values, i.e., when these parameters are both positive or both negative, DNA either lengthens or becomes shorter. We confirmed this behavior and noticed as well that the influence of adjacent nucleotides on helical rise extends over the tetranucleotide, as shown in the two examples reported in table 2. In the first example, tetranucleotide no. 23 (GAGC) is reported; in the second one, tetranucleotide no. 86 (CTGC). In GAGC tetranucleotide, derived from sample 3kbd, we observed an increase in helical rise due to the substitution of an A with a G at the right terminal end and a corresponding change in roll from positive to negative. A similar increase is observed in the CTGC step derived from sample 1bdz. This result suggests that a hexanucleotide code would be more adequate for the evaluation of helical rise than a tetranucleotide code.

**Table 2:** Dependence of helical rise on neighboring bases.

Sample	slide (Å)	roll (°)	sequence	helical rise (Å)
1k9h	-0.26	15.80	TGAGCG	2.34
1k9h	-0.13	28.88	TGAGCG	2.45
1k9l	0.91	8.31	TGAGCG	2.90
1k9l	-0.90	8.36	TGAGCG	2.86
3kbd	-1.30	-6.36	TGAGCA	3.46

1afz	0.46	6.48	CCTGCC	3.42
1cs2	-1.00	0.22	ACTGCT	3.40
1opq	0.44	3.06	TCTGCG	3.31
1bdz	-2.03	-14.18	ACTGCA	4.39
1bdz	-2.18	-16.12	ACTGCA	4.55

Columns report from the left to the right: sample name, slide, roll, sequence and helical rise.

### Symmetric elements in the (H3H4)_2_-tetramer

**Figure 1:**
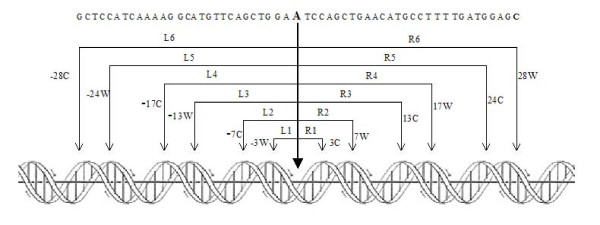
**Central 57 bp of NCP147 nucleosome**. The black thick arrow, from the central, boldfaced Adenine, represents the dyad axis. The sequence is divided into 6 symmetric tracts to the left (L1 to L6) and 6 to the right (R1 to R6), marked by thin arrows. W and C marks the two DNA strands at the DNA-phosphate interaction points with histones in the (H3H4)_2_-tetramer. This frame is referred to as the mask in the text.

The central 57 bp of the nucleosomal particle NCP147 and positions of primary bound DNA phosphates interacting with the (H3H4)_2_-tetramer, mapped by Richmond and Davey [18], are reported in figure 1. The two DNA strands are reported as W and C and the twelve distances L_i _(i = 1, 2 .. 6) and R_i _(i = 1, 2 .. 6) localize DNA segments we measured to assess the presence of a symmetric distribution of lengths. The maximal degree of symmetry Δls (see Methods) is obtained when L_i _= R_i _(i = 1, 2 .. 6). We used the twelve segments reported in figure 1 and the corresponding distances as a mask to compute the probability for each base of a given DNA sequence to represent a dyad axis. The mask in figure 1 covers 57 bp in the sequence and two additional bp are required to calculate the helical rise of the first and the last dinucleotide step of the mask, due to using of the tetranucleotide code. Given, for example, a 100-bp sequence, we compute, using the mask, 41 values starting from the 30^th ^bp up to the 70^th ^bp of the sequence. The translation of the mask along a DNA sequence implies its rotation through about 36°, which is required to follow the helical path of the bases. Under these conditions, in our analysis of nucleosome positioning, translational and rotational phasing are coupled.

Our purpose is to discover DNA sequences in which equal distances are repeatedly inverted, such as in inverted repeats of nucleotides that occur in palindromes. We term these kinds of DNA sequences "palinstases", based on the ancient Greek word "*diastasis*" meaning distance. It is evident that palindromic sequences are palinstasic, but the number of palinstases is expected to exceed the number of palindromes, due to the larger number of possible combinations for 136 helical rise values of tetranucleotides, when compared with the 4 possible DNA nucleotides.

### Symmetric patterns of nucleosomal DNA sequences

**Figure 2:**
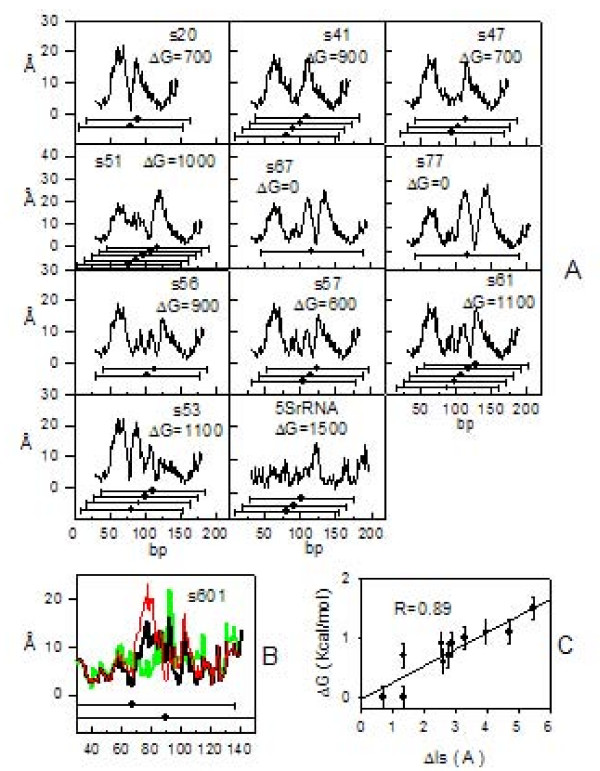
**Nucleosome stability and positioning of synthetic DNA sequences**. A: Δls profiles (black line) of the eleven stable nucleosome-forming DNA sequences reported elsewhere [22]. In the panels, the name of the samples and their ΔG are reported. Dots with horizontal bars (146-bp long) mark positions of mapped nucleosomes. B: Δls profile of s601-sample at different values of helical rise for the step at position 83 of the sequence. The minimal value corresponds to the green curve, the mean value to the black curve and the maximal value to the red curve. C: Scatter plot of nucleosomal stability ΔG vs. degree of symmetry Δls.

We calculated the Δls values (see Methods) for eleven synthetic DNA sequences previously analyzed by Fitzgerald and Anderson [22] in a study of nucleosome translational positioning. These authors mapped nucleosomal positions and determined nucleosomal stability ΔG for each of their samples, including the ΔG value of the nucleosome located on 5S rDNA of Lytechinus variegatus [23]. Results from this analysis are reported in figure 2A. Δls curves exhibit either single, V-shaped, profiles with a minimum pointing towards mapped nucleosomal locations or profiles with multiple minimal Δls values. Such minimal Δls values are always located, with an uncertainty of 10 bp in a few cases, above the positions of mapped nucleosomes, suggesting that their positioning is favored by a symmetric distribution of the distances in relation to the topology of the (H3H4)_2_-tetramer. The variability of Δls values was tested on a sample (s601) originally selected from a pool of synthetic random DNA sequences [24] for its strong nucleosome positioning ability. The presence of two sub-populations of nucleosomes in s601 and their relative abundance in solution, assessed by single-pair fluorescence resonance energy transfer, was previously reported [25]. In figure 2B the two mapped nucleosomal positions in s601 are reported. Δls values (black line) exhibit a minimum that coincides with the first nucleosomal position on the left and several minimal values that are not coincident with the second nucleosomal position on the right. In the center of s601 sequence, at step 83, we noticed the ACGT tetranucleotide (number 110 in table 1), which accommodates the central bistable dinucleotide step CG and has the highest rmsd value (1.35 Å). For this step we substituted the mean value of the helical rise (code value) with minimal and maximal values obtained from the available structures we collected; then we reported the newly generated Δls profiles (green and red line, respectively). This substitution causes changes of about 2 Å in Δls value at the first nucleosomal position and a shift of about 10 bp for the minimum at the second nucleosomal position; this minimum, in new profiles, coincides with the mapped position. These changes can explain minimal Δls values of s67 and s77, whose displacement from mapped dyad positions is probably due to uncertainty characterizing some helical rise values in table 1.

Minimal Δls values in figure 2A, averaged over curves characterized by multiple positions, were plotted as a function of the ΔG value (figure 2C) and a linear relationship, with a correlation coefficient R = 0.89, was obtained. This result indicates that the stability of nucleosomes depends on Δls in a linear fashion and that an increase in Δls destabilizes nucleosomes. Interaction points on the (H3H4)_2_-tetramer and interaction points along the DNA-phosphate backbone can be less or more coincident. DNA can stretch in order to reach a distant interaction point, can increase its curvature in order to interact with a back point or the insertion of bridging water molecules may occur. In fact, X-ray analysis of nucleosomal structure at high resolution showed that, inside the minor groove of DNA strands, up to 121 water-mediated hydrogen-bonds can form [26]. It is evident that the substitution of an electrostatic bond with a weaker hydrogen bond of a bridging water molecule substantially destabilizes the nucleosome.

Thåström et al. [27] reported a sixfold increase in affinity for selected synthetic sequences when compared with the most natural nucleosome positioning. We obtained a similar variation of Δls values (figure 2B) ranging from 0.7 Å, for the most stable synthetic sample, up to 5.5 Å for 5SrDNA, which represents a stable natural nucleosome forming sequence.

The symmetric length-distribution in a given DNA sequence can not be identified in a textual way, i. e., the sequence G40T30G40 is fully symmetric and supposed to have a Δls = 0 at the central TT step. This result seems to be in contrast with the observed low nucleosome positioning affinity of poly-(A/T) tracts. Actually, the Δls profile calculated for this sequence yields a minimum of 2.3 Å, due to differences in helical rise between GGGT, GGTT, GTTT tetranucleotides on the left side and TTTG, TTGG, TGGG tetranucleotides on the right side of the central TT step. It must be mentioned that a Δls = 0 value can be attained by a sequence such as G40T59G40 and the minimum will be located at the central T(30).

We observed very low Δls values (0.3 - 0.6 Å) for synthetic DNA sequences, 150-bp long and characterized by the repetition of the (A/T)3NN(G/C)3NN motif, as well as for (CTG)_50 _bp repeats. It has been shown that these sequences form stable nucleosomes [28,29].

**Figure 3:**
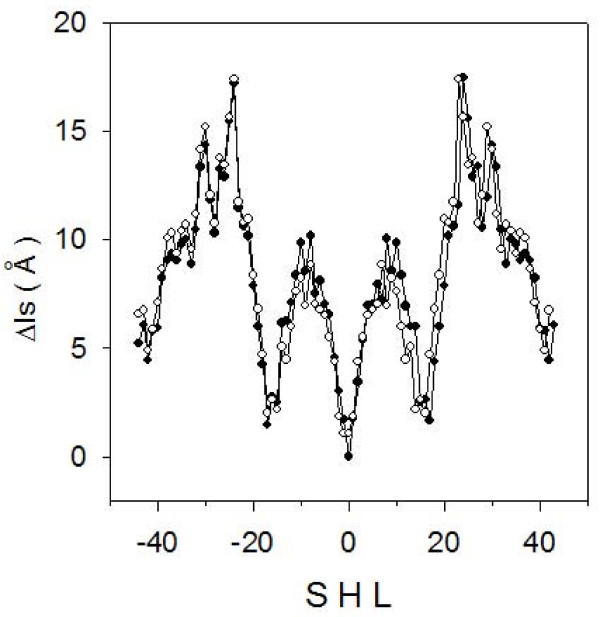
**Symmetric nucleosomal DNA profiles**. Δls profiles of NCP147 (filled circles) and NCP146 (empty circles) as a function of superhelix location (SHL).

We calculated Δls for the sequences of two well characterized nucleosomal particles, NCP147 [18] and NCP146 [30] (figure 3). NCP146 is a 146-bp long palindromic sequence derived from human α satellite DNA, which yields an X-ray structure resolved at 2.8 Å. NCP147 differs from NCP146 by a substitution at position 21 (T → A) and at the corresponding palindromic position 127 (A → T) and by an insertion of a G at position 73. Its X-ray structure is resolved at 1.9Å. This substantial improvement of X-ray diffraction data was obtained upon the increase in the DNA length by just one bp and it was observed that the distribution of interaction points between DNA-phosphates and the histone core was the same for both NCP146 and NCP147. NCP147 is not a perfect palindrome because two dinucleotide steps, AA and AT, located at position -1 and 1, respectively, are not reverse complementary (see figure 1). The sequence becomes palindromic at -2 and 2. Furthermore, by measuring each dinucleotide step with a tetranucleotide code, reverse complementarity is observed only starting from -3W and 3C positions. The measurement of Δ ls values (see Methods) uses the convention of dividing the central segment, from position -3 to 3, into two identical halves in order to make equal the segments L1 and R1 of the mask shown in figure 1. The degree of symmetry of a sequence is, therefore, mainly based on contacts between phosphates and histones beyond positions -3 and 3.

Δls values calculated for the two samples form curves very similar and symmetric with respect to the superhelix location (SHL) 0 of the nucleosomal dyad axis (figure 3). Due to the difference between the two sequences at positions 21 and 127, there are small differences on the left and the right side of the dyad, while a more relevant change occurs at SHL = 0, where NCP146 exhibits two positions with the same Δls value of 1.1 Å in comparison to NCP147, which has a single Δls value close to zero. This difference in the distribution of symmetric distances correlates to the different resolution in X-ray structures obtained for the two particles. The lower Δls value found for NCP147 suggests a higher degree of symmetry and a tighter structure in comparison to NCP146.

### Archaeal nucleosomes

**Figure 4:**
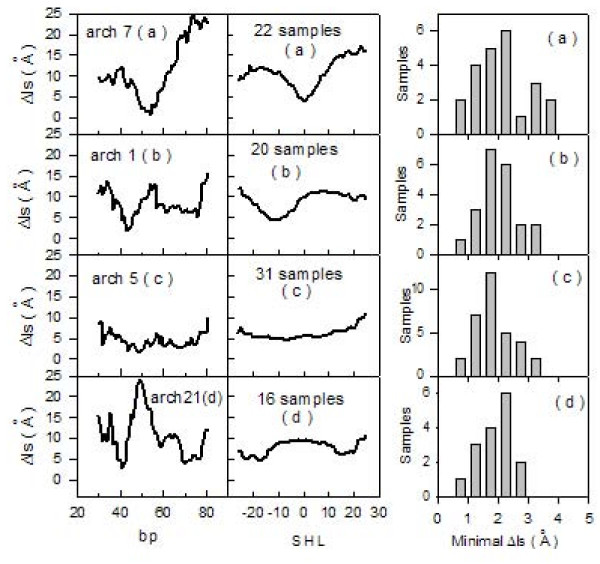
**A: Archaeal nucleosomes**. Left column: Δls profiles (black line). Samples are numbered according to authors' convention [31] and separated into four types: (a), (b), (c) and (d). Central column: Mean Δls profiles (black line) of the four types of archaeal nucleosomes. Right column: Distributions of minimal Δls values for corresponding archaeal profiles.

To further characterize sequences forming stable nucleosomes according to the distribution of Δls values, 89 synthetic sequences, selected for their ability to form very stable archaeal nucleosomes, were analyzed [31]. These particles are usually made of 58 DNA bp bound to archaeal histones and resistant to micrococcal nuclease digestion. Archaeal histones are characterized by the same fold as eukaryotic ones and their quaternary structures resemble (H3H4)_2_-tetramers [32]. The analyzed sequences are 110-bp long and are formed by a variable 60-bp central core and by two identical lateral sequences of 25 bp. We show (figure 4, left column) the calculated Δls values for 4 representative samples, numbered according to authors' convention [31]. All samples were grouped together into four types: 22 samples named (a), with a symmetric profile and a low Δls value located at the center; 20 samples termed (b), with minimal Δls values displaced about 10 bp from the center; 31 samples termed (c), with a region of constant and low Δls values; and 16 samples termed (d), with minimal Δls values displaced by more than 10 bp from the center of the sequence. We report (figure 4, central column) the four mean profiles (a), (b), (c) and (d), obtained by grouping the 89 curves derived from the samples belonging to the same type. The bp number 55 corresponds to SHL 0. The 22 (a)- and the 20 (b)- samples exhibit single symmetrical profiles centered at SHL 0 and -10, respectively. The pattern of the 31 (c)- samples shows tracts that have almost constant Δls values and, consequently, multiple positions for the dyad axis. The 16 (d)-samples have two positions corresponding to minimal Δls values, displaced about 20 bp from the SHL 0 position. Nucleosomal stabilities of all the 89 reported sequences are similar, probably because their recovery was performed at the same step of the purification process [31]. The four mean curves reported in figure 4 (central column) exhibit a common minimal Δls value around 5 Ǻ, which, according to data reported in figure 2, suggests a mean ΔG-value typical of stable nucleosome positioning sequences such a 5SrDNA. Distributions of minimal Δls values for each of the four mean curves are shown on the right of figure 4. It is remarkable that minimal Δls values gather around 2 Å.

### Eukaryotic nucleosomes

**Figure 5:**
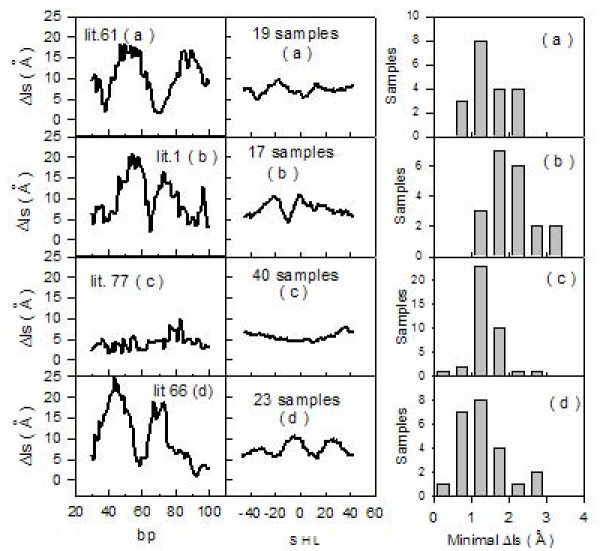
**Nucleosome-forming sequences from literature**. Left column: Δls profiles (black line). Samples are numbered according authors' convention [1] and separated into four types: (a), (b), (c) and (d). Central column: Mean Δls profiles (black line) of the four types of nucleosomes. Right column: Distributions of minimal Δls values for corresponding nucleosomal profiles.

We analyzed 99 sequences of eukaryotic nucleosomes, 146-bp long, cited in scientific literature as strong nucleosome-positioning sequences and with dyad positions mapped at the center of the sequence, as previously reported [1]. We show (figure 5, left column) the calculated Δls values for 4 representative samples, numbered according to authors'convention [1]. All samples were grouped together into four types, according to the same characterization used for archaeal sequences (figure 4). As previously observed for archaeal sequences, minimal Δls values are mainly localized above the center of the sequences. Due to the higher length of these samples if compared to archaeal ones, up to two minimal Δls values, which could represent potential dyad-axis sites, can be observed in the curves at a distance of about 30 bp. The four mean curves (figure 5, central column) exhibit very flat profiles when compared to archaeal analogous curves (figure 4, central column). This is probably due to the uncertainty of ± 20 bp reported in literature for this set of samples [1]. Distributions of minimal Δls values (figure 5, left column) vary, in the four profiles, within the range 1-2 Å ; therefore, we consider these low values as representative of high stability in the considered nucleosomal eukaryotic sequences.

### Asymmetric DNA sequences

**Figure 6:**
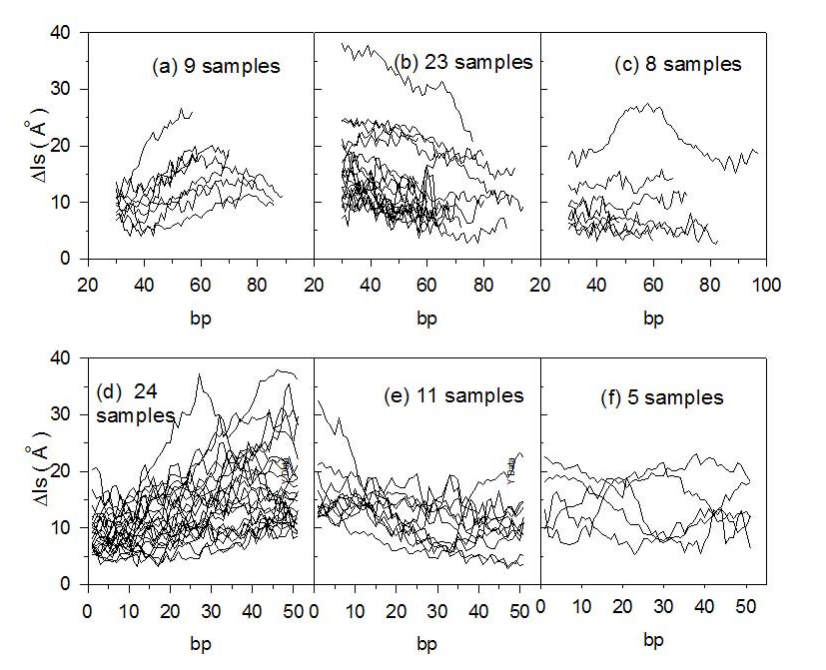
**Nucleosome free sequences**. Δls profiles (black line) for sequences that impair nucleosome formation are shown in panels (a), (b) and (c), while those for sequences accommodating DNase I hypersensitive sites are shown in panels (d), (e) and (f).

We analyzed 40 synthetic DNA sequences selected as refractory to nucleosome formation [33]. These samples range from 86 to 126 bp and the corresponding Δls profiles are reported in figure 6(a)-(c). Further 40 human DNA sequences, 110-bp long, selected for the presence of DNase I hypersensitive sites [34], were analyzed. The associated Δls profiles are reported in figure 6(d)-(f). The studied sequences were grouped according to curve shape: panels (a) and (d) show profiles with an ascending slope, panels (b) and (e) accommodate curves with a descending slope and no slope is present in panels (c) and (f). Most of these sequences differ from those forming stable nucleosomes, which always exhibited minimal Δls values in their center. In the 80 samples which were examined, we observed, in the central part of Δls curves, values ranging from 5 to 30 Å. These values and profiles characterize regions having low affinity for nucleosomes.

### Nucleosomal stability at promoters

**Figure 7:**
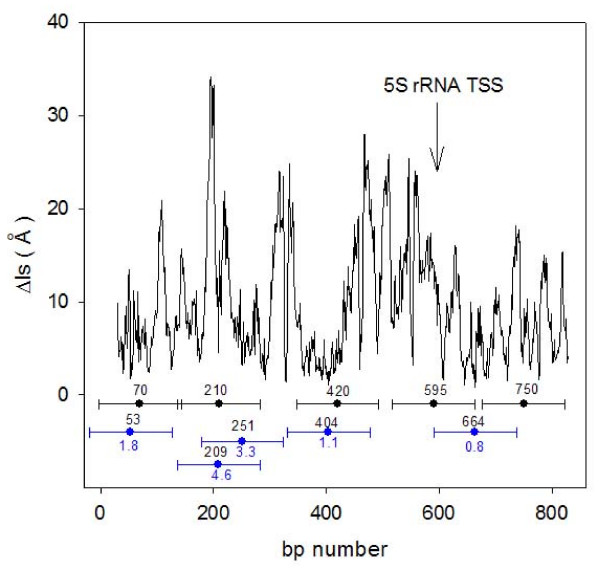
**Localization and stability of 5S rRNA nucleosomes**. Δls profiles (black line) of 869-bp long 5S rDNA sequence from Xenopus borealis. Nucleosomal positions mapped with micrococcal nuclease [35] (black dots in the center of 146-bp long horizontal bars) and mapped according to minimal Δls values (blue dots in the center of 146-bp long horizontal bars) are reported with specific abscissa and ordinate values marked in blue and black digits respectively. The arrow marks the position of the transcription start site.

In figure 7 we report the Δls profile obtained from the DNA sequence of 5S rRNA [35], with published nucleosome positions shown as black dots with 146 bp long horizontal bars. Nucleosome mapping was made with micrococcal nuclease and dyad positions are affected by an uncertainty of ± 20 bp. We assumed the minimal Δls values as possible dyad positions of nucleosomes and marked them with blue dots having 146 bp long horizontal bars in order to visualize the extension of the sequence covered by the nucleosomes. We mapped the nucleosomes starting from the first most stable Δls value of 0.8 Å that is found at position 664 bp. There are several other adjacent minima with similar values around this position that became excluded from the mapping so that the two experimental nucleosomes at position 595 and 750 can not be correctly predicted. We located the second most stable nucleosome with Δls value of 1.1 Å at position 404 bp and the third Δls value of 1.8 Å at position 53 bp. We obtained therefore two coincidences with published positions of 5S rRNA sequence with an uncertainty lower than 20 bp.; a fourth and last nucleosome can be inserted either at position 209 or 251 bp with an uncertainity of 1 bp or 41 bp respectively. We must remember the variability of the Δls profile reported in figure 2B for s601 sample, where minimal Δls values could vary up to 2 Å and shift of about 10 bp in the presence of bistable dinucleotide steps. This reasonably precludes accurate mapping of nucleosomes by use of minimal Δls values. Relative Δls values may instead be assumed as reliable indicators of nucleosomal stability when their measurements are based on a statistical approach. The Δls profile in figure 7 shows, upstream of the transcription start site (TSS), a region of about 200 bp with high Δls values and it is known that nucleosome free regions are always found in proximity of the TSS.

**Figure 8:**
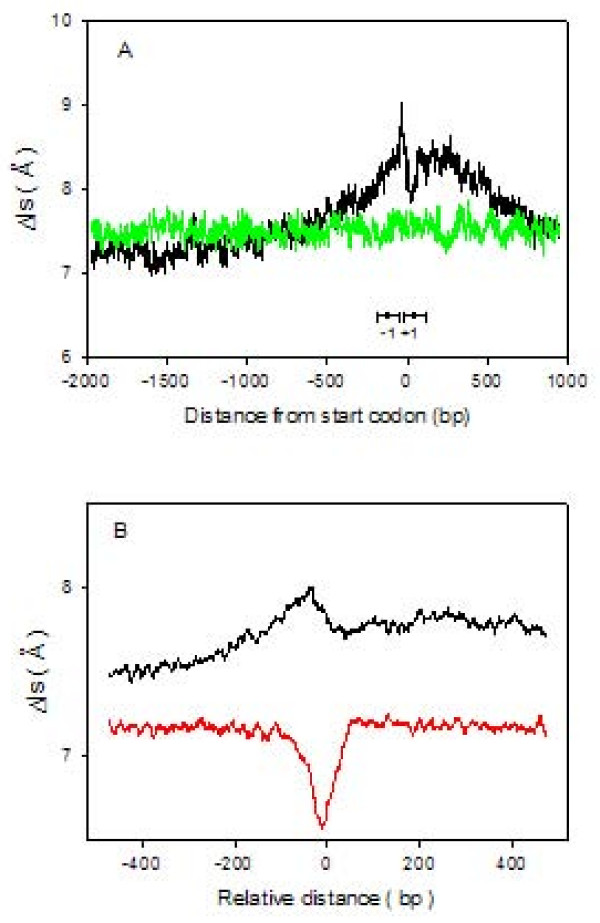
**Nucleosomal stability at promoters**. A: Scatter plot of the mean Δls value averaged over 2126 promoter (black line) and 2126 random (green line) sequences, 3kbp long and aligned at the transcription start site. Black dots with horizontal bars mark mapped nucleosomal positions in the human genome [13]. B: Scatter plot of the mean Δls value averaged over 35650 human promoter sequences at transcription start (black line) and termination (red line) sites.

We analyzed 2126 DNA sequences from promoter regions, experimentally identified, 3 kb in length. They belong to chromosomal genes of vertebrates and represent a set of not-closely-related sequences. We generated 2126 random DNA sequences that were processed the same way for comparison. Mean Δls profiles of promoter and random sequences are presented in figure 8A. A region showing high Δls values is present in the promoter profile if compared to random DNA. Almost 50% analyzed promoters originate from human genome, hence nucleosome positions characterized, in human promoters [13], with -1 and +1, respectively, are reported for comparison. Nucleosome free regions in proximity of the transcription start site have been reported in yeast [36], *Drosophila *[7], and humans [8-13] and, in particular [13], a lower stability of -1 nucleosome, when compared to nucleosome at +1 position, has been observed. According to our model, the highest point of the curve (figure 8A) is the best candidate for representing a nucleosome free region and is located between -1 and +1 nucleosomes at position -37 with respect to the start codon. The upstream region from this point shows a profile with a descending slope and can be considered a weaker nucleosome-forming region. +1 nucleosome is located downstream of the maximal Δls value and positioned under the center of a V-shaped and symmetric profile. The latter resembles many profiles we previously reported as preferred for nucleosome forming. These results indicate a preserved sequence-specificity of nucleosome binding in promoters of vertebrates. The agreement between our results and the experimental mapping obtained by DNA microarray-based methods or computational algorithms supports the consistency of our approach, based on a very simple computation of symmetry. We also analysed DNA sequences belonging to 35650 human promoters [37] 500 bp upstream and downstream with respect to both transcription start and transcription termination site (TTS). Using the same approach reported above a mean profile for each nucleotide was obtained and reported in Figure 8B.

The result related to the region close to the TSS shows the same profile identifying a low affinity for the nucleosomes around 100 bp upstream with respect to the TSS.

A completely different scenario is reported for the region related to the TTS. The plot clearly shows a very high affinity for nucleosome in corrispondence exactly with the predicted TTS. It is remarkable that the extension of the V-shaped plot, corresponding to the potential identified nucleosome, has the extension of about 150 bp, the extension of a nucleosome.

## Conclusion

We guessed that symmetric distributions of DNA lengths could be related to nucleosome formation and suggested two novel ideas to test this hypothesis. First we used a tetranucleotide code in order to measure DNA length and then we searched for symmetric distributions of lengths according to the frame inherent to the concept of *palinstase*. Results previously reported show a linear relationship between nucleosome stability and symmetry measured by Δls values of known nucleosome-forming sequences. Minimal Δls values in the profiles of several analyzed DNA sequences were consistent with preferential nucleosome formation. The presence of many contiguous minimal Δls values (4-5 every 200 bp) and of flat Δls profiles severely limits the use of our results for obtaining genome-wide maps of nucleosome positions. Δls values may instead be assumed as reliable indicators of nucleosomal stability when their measurements are based on a statistical approach. In human promoters we observed low affinity for nucleosome binding at the transcription start site and a high affinity exactly at the transcription termination site. In expectation of the acquisition of more experimental data on DNA helical rise values, we consider our results as a preliminary assessment of the weight of DNA length in nucleosome positioning.

## Methods

We drew on structural databases deposited at http://ndbserver.rutgers.edu/atlas to find helical rise values of naked DNA oligomers obtained by NMR analysis, since this technique suitably applies to samples in the liquid phase, which is more reliable than the crystalline phase to represent the state of DNA in living organisms.

Samples found in the database were selected by discarding those studied in aqueous dilute liquid crystalline phase, which is typically used to resolve long-range structures (> 10 Å), but yields a poor resolution at distances such as those found for helical rise. 99 values of tetranucleotide helical rise, out of the 136 possible ones, were derived this way. 14 further values were found by searching in database samples of DNA oligomers accommodating one modified base when the tetranucleotide sequence of interest was at least two steps away from the modified base. In these samples, we have verified that the presence of the modified base does not change the overall structure of the double helix and checked similarity between helical rise values found either in the modified samples and in the normal ones (data not shown). 10 of the lacking helical rise values were taken from the X-ray database and the remaining 13 were calculated by averaging values for tetranucleotides containing the same central dinucleotide step.

To express the DNA sequence as a linear array of consecutive helical steps, we read the first tetranucleotide of the sequence and derive, from table 1, the first helical rise value related to the dinucleotide step between the second and the third bp. The second tetranucleotide of the sequence yields the value of the helical rise between the third and fourth bp and so on, up to the end of the sequence. Given a sequence of n bp, the number of the elements in the array of helical rise values is equal to n-3. In order to compare positions between various DNA sequences, base-pair numbering coincides with helical-step numbering, but the first helical rise value and the last two ones are lacking. A further decrease in the original number n is due to the use of the mask (figure 1), which covers 56 helical rise values; therefore, the final number of data is n-59.

We compute the rate of symmetry of helical rise distribution for each base pair of any DNA sequence according to the following equation:(1)

where Li and Ri correspond to the lengths shown in Figure 1.

Δls values for the two tracts L1 and R1 are always equal, due to the convention of dividing the central segment from -3 to 3 into two identical halves. The minimal Δ ls value obtained represents the maximum degree of symmetry.

DNA sequences from Archaeal nucleosomes must be requested to:

John N. Reeve at reeve.2@osu.edu

DNA sequences from literature were retrieved from :

http://genie.weizmann.ac.il/pubs/nucleosomes06/segal06_data.html

DNA sequences that impair nucleosome formation must be requested to:

Mikael.Kubista@bcbp.chalmers.se

DNA sequences from DNase I hypersensitive sites were from:

http://www.research.nhgri.nih.gov/DNaseHS/May2005/

DNA promoter sequences of vertebrates were retrieved from the EPD database:

http://www.epd.isb-sib.ch/seq_download.html

DNA promoter sequences of human genome were from :

http://genome.ucsc.edu/ENCODE/encode.hg17.html

## Authors' contributions

FP leaded the project, designed the computational analysis and drafted the initial manuscript. DS performed the computational analysis and helped to draft the manuscript. All authors read and approved the final manuscript.
